# Supplementary material for: Transcriptome and Metabolome Analyses Provide Insights into the Watercore Disorder on “Akibae” Pear Fruit
Source: Int J Mol Sci. 2021 May 6;22(9):4911. doi: 10.3390/ijms22094911 (PMC8124519; doi:10.3390/ijms22094911)
Supplement: Supplementary file 1 [file ijms-22-04911-s001.zip › Table S1.pdf]

Table S1. List of primers

ID	Forward	Reverse
Chr12.g35872	GAGCGCTGCAAATCGGAAAT	TCGCAACATGGAGCAAAAAGA
Chr16.g29529	CATTTGTGCACTCCCACAGC	TGCTTTTGGATCGAGCACCT
Chr15.g02360	GGCCATGGTCCTTCCATCAA	CCTACAGTGCGGCCATGTTA
Chr16.g30736	GCCGTGAGAGTACTCAGCAAA	TGCGCTTAAACCCCATTTTGG
Chr4.g40405	TGTTCTTCCTTCTACCCTTTCTACT	AGTTCACTGCTTAGCTCAGTT
Chr5.g06254	CATGAAGGTGCCCGAACATC	TAATGGTTGCCCATCCAGCC
Chr3.g18628	AGCAAAGCTACACCCTGTCA	CATGTTGATTGAGCCGGTCC
Chr8.g55784	TTGATCCTGCTGGATGGCTT	AGTGTTTGATCCAGGACCAGT
Chr11.g13340	TACCGACGCCGCTAATTTCA	CGGTGTAAGGCACTCCTGTT
Chr8.g54319	TCAGAGGCTGCTGGTTCTTG	ACCCAGATGCCTTCACTTGG
