# Supplementary figures and images for: Transcriptome and Metabolome Analyses Provide Insights into the Watercore Disorder on “Akibae” Pear Fruit
Source: Int J Mol Sci. 2021 May 6;22(9):4911. doi: 10.3390/ijms22094911 (PMC8124519; doi:10.3390/ijms22094911)

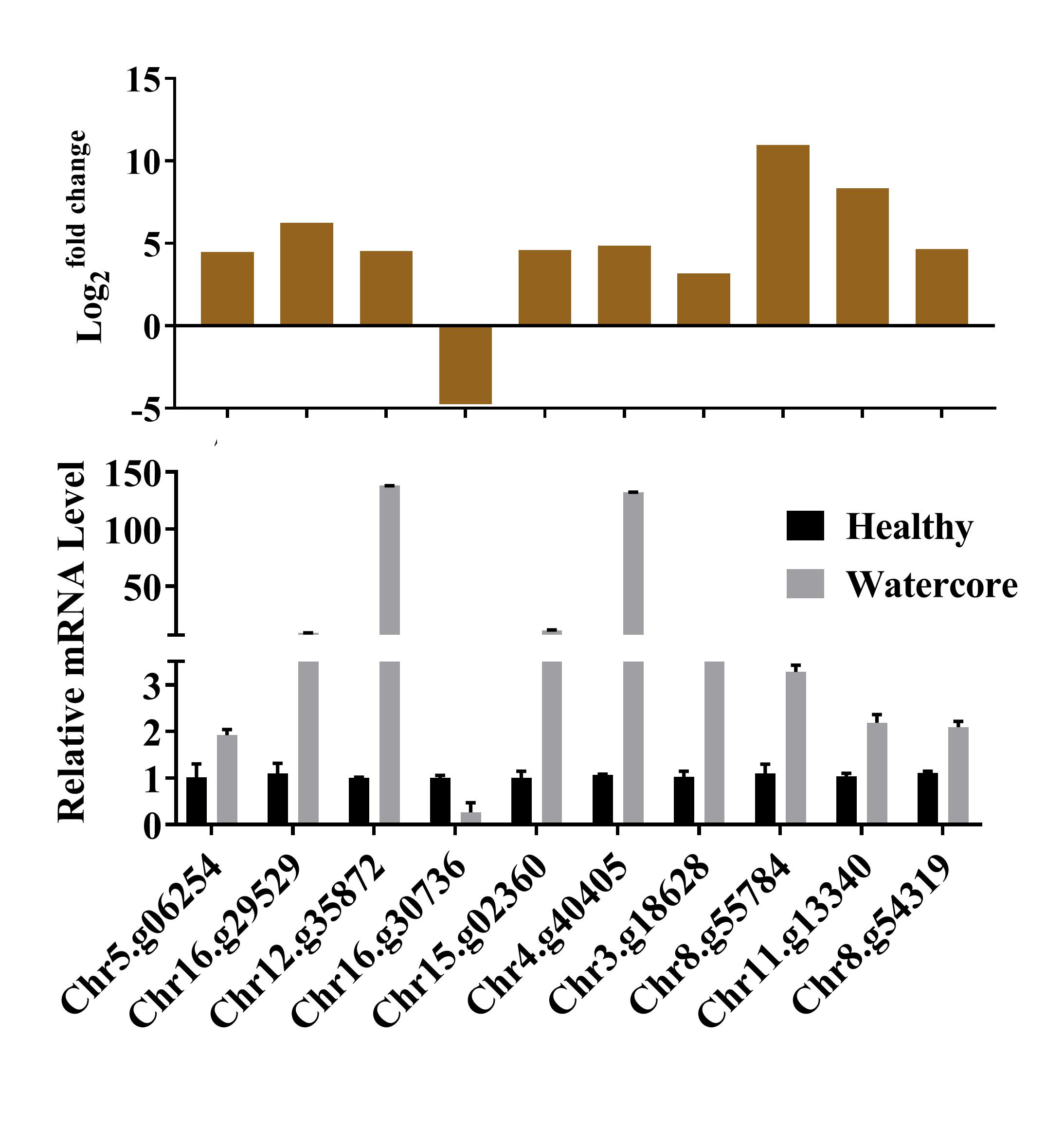

Supplement: Supplementary file 1 [file ijms-22-04911-s001.zip › Fig.S1.jpg]

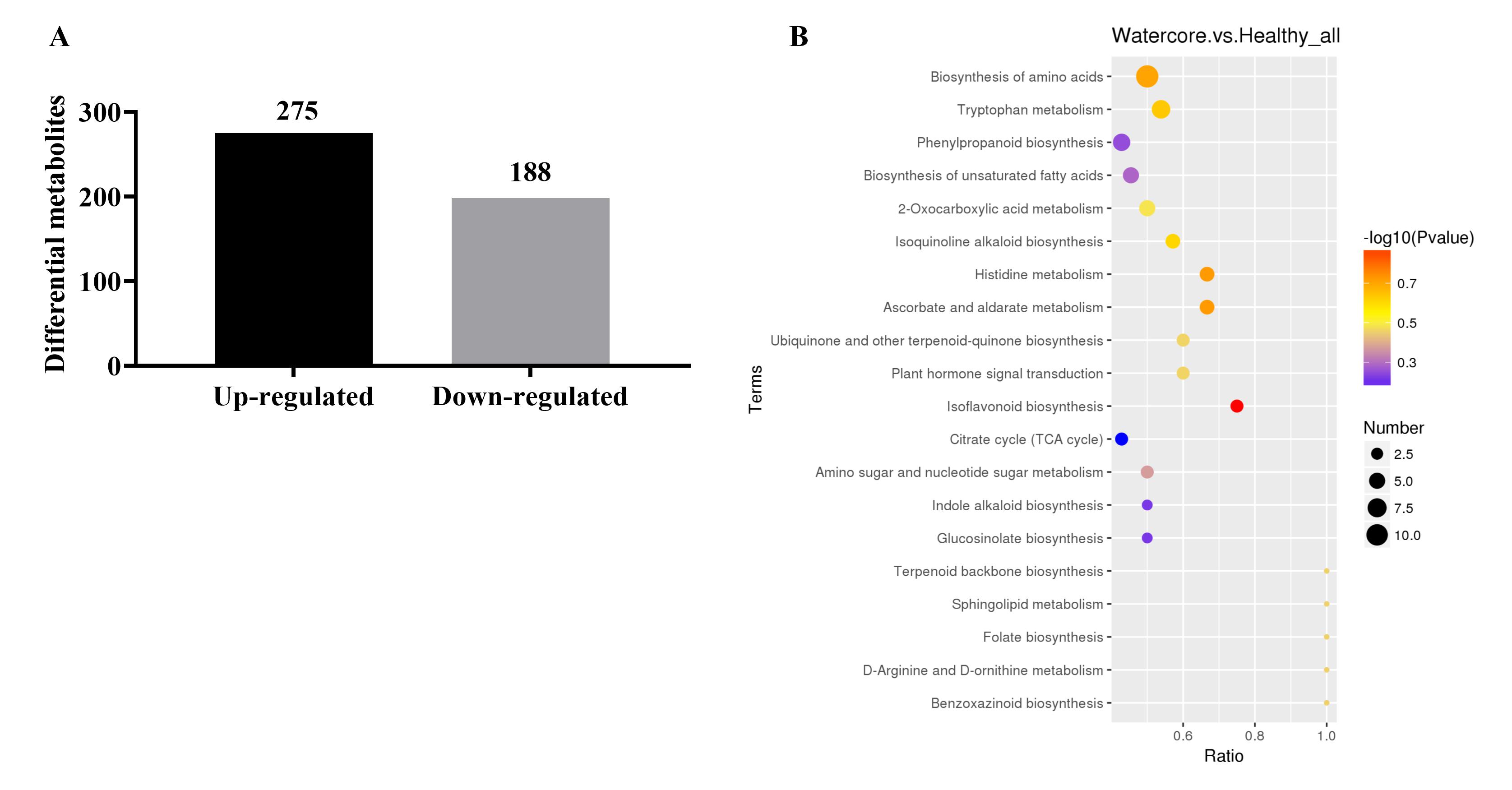

Supplement: Supplementary file 1 [file ijms-22-04911-s001.zip › Fig.S2.jpg]
